# Rhythmic and melodic deviations in musical sequences recruit different cortical areas for mismatch detection

**DOI:** 10.3389/fnhum.2013.00260

**Published:** 2013-06-07

**Authors:** Claudia Lappe, Olaf Steinsträter, Christo Pantev

**Affiliations:** ^1^Department of Medicine, Institute for Biomagnetism and Biosignalanalysis, University of MünsterMünster, Germany; ^2^GSI Helmholzzentrum für Schwerionenforschung GmbHDarmstadt, Germany

**Keywords:** music perception, mismatch negativity, beamforming, cortex

## Abstract

The mismatch negativity (MMN), an event-related potential (ERP) representing the violation of an acoustic regularity, is considered as a pre-attentive change detection mechanism at the sensory level on the one hand and as a prediction error signal on the other hand, suggesting that bottom-up as well as top-down processes are involved in its generation. Rhythmic and melodic deviations within a musical sequence elicit a MMN in musically trained subjects, indicating that acquired musical expertise leads to better discrimination accuracy of musical material and better predictions about upcoming musical events. Expectation violations to musical material could therefore recruit neural generators that reflect top-down processes that are based on musical knowledge. We describe the neural generators of the musical MMN for rhythmic and melodic material after a short-term sensorimotor-auditory (SA) training. We compare the localization of musical MMN data from two previous MEG studies by applying beamformer analysis. One study focused on the melodic harmonic progression whereas the other study focused on rhythmic progression. The MMN to melodic deviations revealed significant right hemispheric neural activation in the superior temporal gyrus (STG), inferior frontal cortex (IFC), and the superior frontal (SFG) and orbitofrontal (OFG) gyri. IFC and SFG activation was also observed in the left hemisphere. In contrast, beamformer analysis of the data from the rhythm study revealed bilateral activation within the vicinity of auditory cortices and in the inferior parietal lobule (IPL), an area that has recently been implied in temporal processing. We conclude that different cortical networks are activated in the analysis of the temporal and the melodic content of musical material, and discuss these networks in the context of the dual-pathway model of auditory processing.

## Introduction

Playing a musical instrument brings together the faculties of the human body in the most intricate way. It involves the translation of visual cues from the notation, fine auditory discrimination, precise control of complex movements, and detailed somatosensory feedback about their execution. It requires a major effort to coordinate the multitude of processing in a manner adequate for pleasing performance. Indeed, musicians have to have talent and have to exercise immensely to achieve good levels of control over their instrument. Many studies have shown that the brains of musicians exhibit structural and functional differences compared to non-musicians (Münte et al., 2002; Fujioka et al., 2004; Herholz et al., 2009). Musicians show for example a stronger auditory cortical representation than non-musicians for tones of the musical scale and for the timbre of the instrument on which they were trained (Hirata et al., 1999; Pantev et al., 1998, 2001; Shahin et al., 2003). Musicians show furthermore differences in gray matter volume in motor, auditory and visual brain regions as compared to non-musicians (Schneider et al., 2002; Gaser and Schlaug, 2003). To study musical expertise with EEG (electroencephalography) or MEG, researchers have used the musical mismatch negativity (MMN) (Fujioka et al., 2004; Herholz et al., 2008; Lappe et al., 2008, 2011). The MMN is an event-related potential (ERP) response representing the violation of a learned acoustic regularity (Näätänen and Alho, 1995). It is considered as a pre-attentive change detection mechanism at the sensory level on the one hand and as a prediction error signal on the other hand, suggesting that sensory information is matched with top-down predictions (Garrido et al., 2009). In musicians, the musical MMN, which is elicited in musical material, is stronger than in non-musicians (Fujioka et al., 2004).

## Musical training

When comparing musicians to non-musicians it is difficult to separate the effects of training and talent. It is very likely that years of training have left their mark on a musician's brain, but it is also conceivable that those with higher musical talent advance further in their musical career. To study the effects of musical training directly requires to monitor training effects in non-musicians that begin to receive musical training. A few of such studies have been undertaken. They have shown fast training effects on brain responses. In a study by Bangert and Altenmüller (2003) subjects learned to associate keypresses with musical sounds while a control group received random allocations of sounds to keypresses preventing such a learning. Auditory-motor co-activation in EEG recordings was observed after training in the experiment group already after 20 min. This effect was enhanced after 5 weeks of piano training.

**Figure 1:**
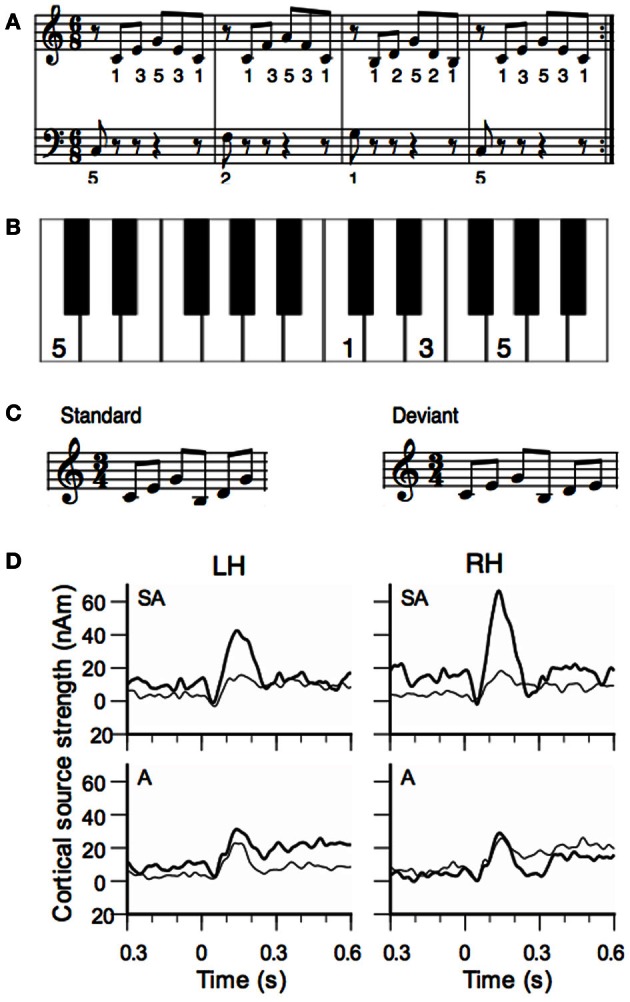
**(A)** Training material of the study of Lappe et al. (2008): the piano exercise consisted of four broken chords forming the I-IV-V-I chord progression in C-major. **(B)** To facilitate training a template was used where the image of the keyboard was depicted and where the finger placement was marked. Panel **(B)** shows the template of the first measure. **(C)** Stimuli for the MEG measurement before and after training. The standard stimulus consisted of a C-major and a G-major broken chord in first inversion. In the deviant the last tone was lowered by a minor third. **(D)** Source waveforms of the MMN response to deviant stimuli before and after training. Data of the sensorimotor-auditory group (SA-group) are depicted in the top, data from the auditory group (A-group) in the low row. Thin lines show pre-training, thick lines show post-training data. The left hemisphere is depicted in the left (LH), the right hemisphere in the right column (RH).

Lappe et al. (2008) investigated short-term training effects by means of the musical MMN. A group of non-musicians learned to play a melody sequence (Figure 1A) on the piano for eight sessions distributed over two weeks. To facilitate training a template was used where the image of the piano was depicted and where the finger placements were marked (Figure 1B). Before and after piano training the subjects' responses to a musical MMN were recorded with MEG. The test sequence of six tones consisted of a G-major broken chord which was followed by a C-major broken chord. In the deviant condition the last tone was lowered by a minor third (Figure 1C). After training the MMN was significantly increased (Figure 1D). The enhancement was stronger in the right hemisphere than in the left hemisphere.

## Sensorimotor interaction

This experiment showed that short-term musical training increases the musical MMN as an indicator for musical expertise. However, the experiment also showed that sensorimotor interactions are important to achieve the training effect. This was evidenced by a second group of non-musicians that received only auditory training but did not play themselves. Each subject of this group (the auditory or “A” group) was matched to one of the piano-trained subjects (the sensorimotor-auditory or “SA” group) and listened to all of the training sessions of that subject. During auditory training, subjects of the A-group were seated in front of the piano, but they received no visual information about the keys that had been pressed. Participants of the A-group had to press the right or left foot-pedal to indicate that the sequence they heard was correct or not. This was done to ensure that subjects of the A-group participated actively and listened carefully. The assignment of the test subjects to either the SA or to the auditory (A) group was random. The results showed no improvement of the MMN responses after auditory training (A-group, Figure 1D, lower row), in contrast to the SA-group. Thus, the sensorimotor interactions that occurred during the piano training in the SA-group, in which hand movements and tone generations are paired, were necessary for the training effect to be established in the MMN.

Evidence for the importance of sensorimotor interaction in musical training are also seen in the aforementioned study by Bangert and Altenmüller (2003). Plasticity after short-term piano training occurred in the right hemispheric areas including the auditory and motor regions. A related fMRI study showed significant activation in Broca's area and the adjacent ventral pre-motor cortex (vPMC) when listening to previously learned stimuli but not when participant listened to familiar but untrained melodies (Lahav et al., 2007).

## Melody and rhythm

**Figure 2:**
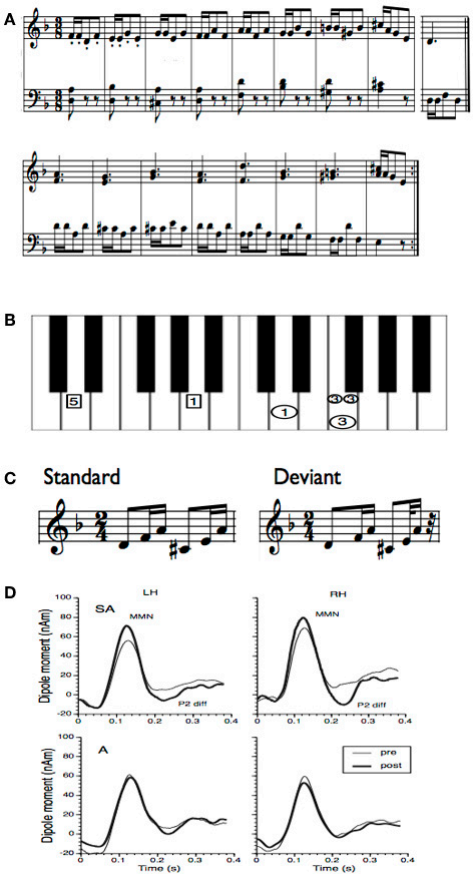
**(A)** Training material for the rhythm study of Lappe et al. (2011). The training material was taken from a piano exercise book (Proksch M, 2000) (Proksch, 2000). **(B)** To facilitate training a template was used where the image of the keyboard was shown. In this figure only one measure is depicted. Small circles indicated that notes should be played at double speed. In general the circles marked that the right hand should be used, the rectangles indicated to use the left hand. All numbers depicted in one horizontal line had to be played simultaneously. **(C)** Stimuli for the MEG measurement before and after training. In the deviant the last tone was presented 100 ms earlier. **(D)** Group averages of the source waveforms obtained after performing source-space projection before and after training for both groups and hemispheres. Thin lines indicate pre-training and thick lines post-training data. The left column depicts the source waveform of the left, the right column depicts the source waveform of the right hemisphere.

Besides melody, rhythm is another basic feature of music. Musical rhythm is based on a recurring pulse or meter, which makes musical events highly predictable. Tapping to a beat is a natural behavior and for most people there is no specific musical training necessary to correctly predict upcoming musical events (Zatorre et al., 2007; Geiser et al., 2009). However, musicians are more accurate in the perception and production of rhythm and meter than non-musicians (Geiser et al., 2010). The ability of musicians to better discriminate differences in rhythmic timing is reflected in an enhanced MMN generated by a rhythmic deviation (Vuust et al., 2005). Like for melody, a short period of piano training in non-musicians induces an increase in the MMN to rhythmic deviations (Figure 2) (Lappe et al., 2011). The ERP results (Figure 2D) showed a significant enhancement of the MMN only for the participants that actively played during the training (SA-group), not for participants that underwent only auditory training (A-group). Furthermore, the brain activation for rhythmic deviations was bilateral whereas the activation for pitch deviants (Figure 1) was significantly stronger in the right hemisphere.

## Brain structures for musical deviance detection

Studies with frequency or duration change in a sequence of non-musical stimuli have localized the generators of the MMN mainly in the superior temporal gyrus (STG) and, to a lesser extent, in areas of the inferior frontal cortex (IFC) (Rinne et al., 2000; Waberski et al., 2001; Opitz et al., 2002; Molholm et al., 2005; Schönwiesner et al., 2007; Tse and Penney, 2008) [for an overview see Deouell (2007)]. The superior temporal activation is believed to reflect auditory deviance detection at the sensory level. The contribution and functional role of the IFC is still a matter of debate. For musical material, the early right anterior negativity (ERAN), an ERP response similar to the musical MMN that is elicited about 150 ms after music syntactic irregularities (harmonically inappropriate chords) (Koelsch et al., 2001), was localized using equivalent current dipole (ECD) analysis. It revealed a neural source region near Broca's area and its right hemisphere homolog (Maess et al., 2001). Since Broca's area is also involved in syntactic aspects of language comprehension, it was suggested that harmonic-syntactic violations in musical material is processed in similar neural networks (Koelsch et al., 2001; Maess et al., 2001). The neural correlates of processing harmonically related and unrelated musical sounds were also investigated in an fMRI study by Tillmann et al. (2003). In this study IFC showed stronger activation in response to unrelated compared to related musical sounds (Tillmann et al., 2003). These experiments provide evidence that the IFC, as part of the anterior-ventral auditory pathway, is involved in a more detailed analysis of auditory object identification (Rauschecker and Scott, 2009).

## Localizing MEG sources with beamforming

MEG has a high temporal resolution, and, in contrast to the delay of the hemodynamic response in functional MRI, enables measuring brain activity within a time resolution of milliseconds. Furthermore, the recorded brain magnetic fields in MEG are less distorted by the skull and scalp as compared to EEG, thus allowing a more accurate spatial resolution. MEG is therefore an adequate method for localizing activation areas and investigating temporal resolution of auditory processing.

Beamforming, or Synthetic Aperture Magnetometry (SAM) is a method of source analysis of MEG sensor data in which a spatial filter is used to estimate the contribution of a given source location to the measured MEG sensor signal, while filtering out the contributions of other sources (Robinson and Vrba, 1998). The advantage of this method is that it is not necessary to impose constraints on the source solution by determining the number and positions of the ECDs in advance (Hillebrand et al., 2005; Steinsträter et al., 2010). Since beamformer reconstructions of the source wave forms of signals originating from highly time correlated sources may be heavily distorted (Sekihara et al., 2002), the sensors of the two hemispheres should be processed separately for a beamformer analysis of auditory signals (Herdman et al., 2003).

The beamformer approach SAM can be applied together with pseudo-*T*-value statistics (Robinson and Vrba, 1998). Beamformers output basically the variance of a signal of a current dipole at a given brain location across a given time window. Pseudo-*T*-values describe the contrast in signal strength between an “active state,” and a “control state.” In order to increase the depth resolution this difference is normalized by an estimation of the sensor noise (singular value decomposition of the data covariance matrix) and spuriously mapped by the beamformer to the brain.

This method has originally been developed to separate an active, task related condition, from the background brain activity (Robinson and Vrba, 1998), but can be used to contrast the deviant against the standard stimulus in an MMN design.

The beamformer produces a volumetric image of signal contrasts (pseudo-*T*-values), here with a spatial resolution of 3 mm, which is similar to the activation maps known from fMRI studies. Then, SPM can be used for the group analysis and the spatial normalization for the subjects (averaged across runs). Since one cannot assume a normal distribution for volumetric maps of pseudo-T images, non-parametric permutation tests should be used to investigate the significance of activated brain regions (Nichols and Holmes, 2001).

## Beamformer localization of MMN to melodic deviants

**Figure 3:**
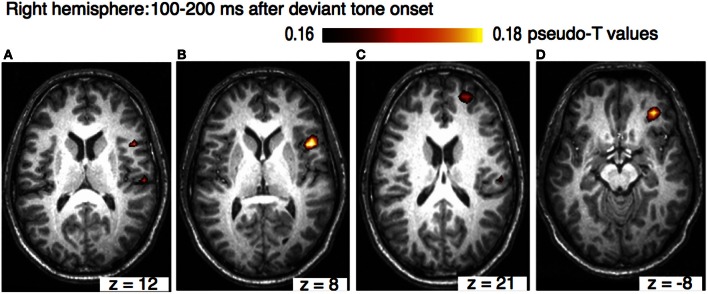
**Axial view of the right hemisphere (overlaid on an individual anatomical MRI) showing significant activations (pseudo-*T*-values, significance level 5%) of the musically elicited mismatch negativity within a time window of 100–200 ms after the occurrence of a pitch deviation.** Panel **(A)** shows activation in the right STG (MNI coordinates: *x* = 62, *y* = −20, *z* = 16). Panel **(B)** shows activation in IFC (MNI coordinates: *x* = 51, *y* = 12, *z* = 8), panel **(C)** shows activation SFG (MNI coordinates: *x* = 25, *y* = 55, *z* = 21), and panels **(D)** shows activation in OFC (MNI coordinates: *x* = 35, *y* = 42, *z* = −8).

**Figure 4:**
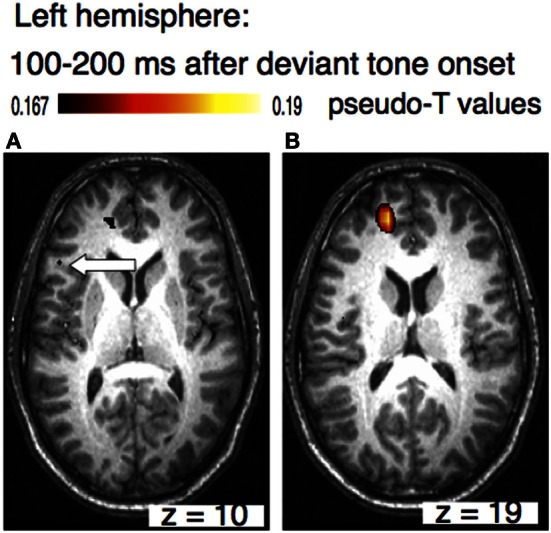
**Axial view of the left hemisphere of the musically elicited mismatch negativity within a time window of 100–200 ms after the occurrence of a deviant tone.** Panel **(A)** shows activation within the triangular part of the inferior frontal cortex (MNI coordinates: *x* = −51, *y* = 25, *z* = 10, left arrow), panel **(B)** depicts activation within superior frontal cortex (MNI coordinates: *x* = −20, *y* = 52, *z* = 19).

The sources of the MMN to melodic deviants were localized in a beamformer analysis (Lappe et al., 2013). This analysis was performed on the post-training data of the SA-group from the melodic MMN study (Lappe et al., 2008) within the time window of 100–200 ms, i.e., at the peak of the MMN response (Figure 1D). The analysis revealed significant neural activation in the superior temporal (STG), the IFC, and the superior frontal (SFG) and the orbitofrontal gyri (OFG) in the right hemisphere (Lappe et al., 2013) (Figure 3). IFC and SFG activation was also observed in the left hemisphere (Figure 4). These results suggested that immediately after the occurrence of a deviant tone a distributed network was activated in right hemispheric auditory cortices, and bilaterally in inferior frontal and prefrontal areas. This gives further evidence that a pitch or harmonic deviation within musical material activates a neural network comprising auditory cortices spreading anterolaterally into inferior and prefrontal areas. The result is in line with the notion that auditory object recognition is supposedly processed in the ventral part of the auditory pathway (Rauschecker and Scott, 2009).

The pronounced activation of IFC might also be due to musical training, since neurons of the vPMC, that are located adjacent to Brodmann area 44, might have contributed to the musical MMN. Piano training might have established an internal forward model linking a specific motor movement with an auditory sound (Lee and Noppeney, 2011). It is possible that such a model supported predictions about upcoming events and enabled a more precise concept about upcoming tones. An internal model involving the prefrontal areas might help to detect easier auditory prediction violations, as manifested by the musical MMN.

## Beamformer localization of MMN to rhythmic deviants

A dissociation between melodic and rhythmic processing was observed in a study in which musicians played melodic and rhythmic focussed musical sequences during an MRI scan (Bengtsson and Ullen, 2006). In that study melodic information was processed in the medial occipital lobe, the superior temporal lobe, the rostral cingulate cortex, the putamen, and the cerebellum. In contrast, rhythmic information was processed in the lateral occipital and the inferior temporal cortex, the left supramarginal gyrus, the left inferior, and ventral frontal gyri, the caudate nucleus, and the cerebellum This study, however, was not a mismatch study and the results obtained from this experiment showed a cross-modal effect since subjects played from a visually displayed score. Lesion studies, on the other hand, found that patients with a temporal lobe damage were impaired in melody processing (Alcock et al., 2000), while rhythmic processing was impaired after a left temporo-parietal lesion (DiPietro et al., 2004). Imaging studies investigating the processing of rhythm perception have also shown activation in the basal ganglia, the insula, left inferior parietal lobule (IPL) (Bamiou et al., 2003; Limb et al., 2006; McAuley et al., 2012), and auditory and motor regions (Chen et al., 2006; Grahn and Brett, 2007).

Vuust et al. (2005) performed a dipole analysis to localize the MMN elicited by a rhythmic deviation in musical material. They found bilateral activation in the vicinity of auditory cortices with a left-laterality effect in musicians (Vuust et al., 2005). However, to investigate the possible contributions of other areas requires a distributed source model. We therefore conducted beamforming analysis on the post-training data of our rhythm MMN study (Lappe et al., 2011). To introduce this analysis we need to describe in more detail the methods and data of that study (Figure 2).

Ten non-musician subjects with normal hearing between 24 and 38 years old were trained to play a rhythm focussed piano exercise (Figure 2A) within 2 weeks comprising eight training sessions, each lasting 30 min. Informed written consent was obtained from all subjects to participate in the study. The study was approved by the Research Ethic Board of the University of Münster. For sensorimotor training a template was used where the keys, the tempo of the tones and the finger placement were marked, so that participants did not have to learn the musical notation before training (Figure 2B). Plasticity effects were measured by means of the musical MMN. The stimuli were generated with a digital audio workstation including an integrated on-screen virtual keyboard which permitted the generation of realistic piano tones on a synthesized piano. Stimuli consisted of a d-minor broken chord in root position d′-f^′^-a′ followed by an A-major chord in first inversion: c sharp-e′-a′ (Figure 2C). The standard stimulus was composed of two identical rhythmic figures, beginning with an eighth note (400 ms) and followed by two sixteenth notes (200 ms each) resulting in a total stimulus length of 1600 ms. On deviant trials, the sixth tone was presented 100 ms earlier leading to a total stimulus length of 1500 ms. Successive sequences were separated by a silent interval of 900 ms. Sequences were presented in two runs, each run comprising 320 standards and 80 deviant trials presented in a quasi random order.

A 275-channel whole-head magnetometer system (Omega 275; CTF Systems) was used to record the magnetic field responses. The recordings were carried out in a magnetically shielded and acoustically silent room. MEG signals were low-pass filtered at 150 Hz and sampled at a rate of 600 Hz. Epochs contaminated by muscle or eye blink artifacts containing field amplitudes that exceeded 3 pT in any channels, were automatically excluded from the data analysis. Subjects were seated in an upright position as comfortable as possible while ensuring that they did not move during the measurement. Subjects were instructed to stay in a relaxed waking state and not to pay attention to the stimuli. To distract attention from the auditory stimuli subjects watched a soundless movie of their choice, which was projected on a screen placed in front of them.

The subject's head position was measured at the beginning and at the end of each run by means of three localization coils that were fixed to the nasion and to the entrances of both ear canals (fiducial points).

A T1-weighted MR image was obtained from each participant using a three Tesla Scanner (Gyroscan Intera T30, Philips, Amsterdam, Netherlands). Turbo Field acquisition was applied to collect 400 contiguous T1-weighted 0.5-mm thick slices in the sagittal plane. For co-registration with the MEG measurements the positions of the fiducial points (filled with gadolinium to be visible in the MRI) were used.

Epochs of 3.1 s (comprising one standard and on deviant stimulus) were extracted from the dataset and filtered by 1–30 Hz bandpass filter. A multi-sphere head model fitted to the individual participants' structural MRI was used as volume conductor. To achieve as much similarity as possible between the background brain activity during the active and control condition, we contrasted each deviant with its directly preceding standard. This reduces the number of analyzed standards to the number of deviants (80; for each run 80 × 2 × 2 = 320 epochs). We conducted the beamforming analysis within the time window of 100–200 ms. The time window was chosen according to the corresponding source wave form that were obtained from the ERP analysis (Figure 2D). The activation window was contrasted, as described earlier, to the corresponding standard time window.

**Figure 5:**
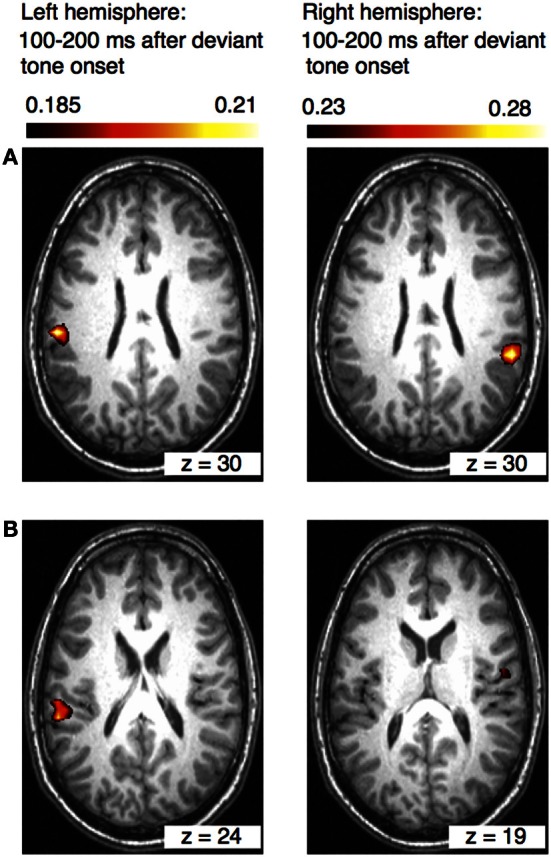
**Axial views of the left and right hemispheres (overlaid on an individual anatomical MRI) showing significant activations (pseudo-*T*-values, significance level 5%) of the musically elicited mismatch negativity within a time window of 100–200 ms after the occurrence of a rhythmic deviation.** Panel **(A)** shows activation within the left and right inferior parietal lobule (IPL) (MNI coordinates left: *x* = −58, *y* = −27, *z* = 30; right: *x* = 59, *y* = −41, *z* = 30). Panel **(B)** shows activation within the left superior temporal gyrus (STG) and in the right insula (MNI coordinates left: *x* = −60, *y* = −34, *z* = 24; right: *x* = 59, *y* = 41, *z* = 19).

The results of the beamformer analysis can be seen in Figure 5. Significant neural activation occurred in temporal and parietal cortex.

## Different brain networks for melodic and rhythmic deviance detection

Comparing the beamformer results between the melody and the rhythm data sets shows that pitch and rhythm deviations are processed in different brain areas. While a melody deviation elicited activation in the right STG, in the right OFC, in the IFC, and SFG bilaterally, rhythmic deviation elicited activation in the left STG, the left and right IPL, and the right insula. One has to remember, however, that the results were obtained in a between-group and not in a within-subject design. However, the design of the two studies was identical except for the musical material that was trained. The results confirm previous music studies showing similar activation areas after a harmonic or rhythmic deviation (Maess et al., 2001; Tillmann et al., 2003; Vuust et al., 2005).

The beamformer analysis indicates that the differential processing of melody and rhythm is reflected in the musical MMN. Differential processing of melody and rhythm is in accordance with the dual-pathway model of auditory processing (Belin and Zatorre, 2000; Zatorre et al., 2007; Rauschecker and Scott, 2009). According to the dual pathway model, the antero-ventral stream projects ventrally to anterior, inferior frontal and prefrontal areas. This processing stream is presumably important for auditory pattern and object recognition. The postero-dorsal pathway on the other hand projects to parietal areas and has been associated with the processing of space and time (Bueti and Walsh, 2009). The activation in the right hemisphere in STG, OFC, and bilaterally in IFC and SFC in our melody study suggests that pitch deviations are processed in the ventral path of auditory pathways. These results corroborate the findings of studies investigating musical expectancy violation after a syntactic or pitch related deviation within a musical sequence that demonstrated auditory and IFC activation (Maess et al., 2001; Koelsch et al., 2002; Tillmann et al., 2003; Koelsch, 2005).

Whereas superior temporal activation in auditory deviance detection is generally associated with sensory processing, the role of inferior frontal gyrus using this experimental paradigm is still a matter of debate. In some studies IFC activation in auditory novelty processing has been associated with attentional mechanisms (Schönwiesner et al., 2007). Other studies investigating the role of IFC in auditory deviance detection with non-musical material have demonstrated that IFC activation increases when auditory deviance decreases linking the IFC to a contrast enhancement mechanism which could indicate that IFC supports the STG system to discriminate auditory stimuli (Opitz et al., 2002; Doeller et al., 2003). The aforementioned studies investigating auditory deviance detection in musical material have shown on the other hand that neural activation in that brain area is stronger for harmonically unrelated as compared to related tones (Tillmann et al., 2003). It has furthermore been demonstrated that the amplitude of the ERAN, which has been localized in BA 44 as part of IFC, depends on the degree of harmonic appropriateness (Koelsch et al., 2001; Maess et al., 2001). In addition, a previous voxel-based morphometry study demonstrated a reduction in white matter concentration in the right IFC in amusic subjects with severely impaired pitch discrimination ability. Subjects were classified as amusics based on the Montreal Battery of Evaluation of Amusia (MBEA) (Hyde et al., 2006). The results of these studies suggest that the IFC as part of the ventral auditory pathway is indeed crucial for auditory object recognition.

Expectancy violation to a rhythmic progression within a musical sequence, on the other hand, seems to be processed in the posterior part of STG and the IPL. The significant neural activation of the IPL within 100–200 ms after the occurrence of rhythmic deviation also fits to the dual stream model of auditory processing. The postero-dorsal pathway connects the posterior part of auditory cortex with parietal areas and is associated with neural networks processing time varying events (Rauschecker and Scott, 2009). The parietal lobe plays indeed an important role in processing temporal and spatial information, in integrating sensory information from different modalities, and in performing sensorimotor transformations for action. Temporal and spatial information for example, which is required for planning subsequent motor behavior, is integrated within the parietal lobe (Bueti and Walsh, 2009). The posterior part of auditory cortex and the IPL as part of the dorsal stream are also crucial for sensorimotor auditory transformations (Warren et al., 2005). The strong connection between auditory and motor systems in the time domain is evident in music (Zatorre et al., 2007; Geiser et al., 2009, 2010). Synchronizing movements to musical beats is a common human behavior. Functional connectivity between posterior STG and dPMC during a rhythmic tapping task was previously demonstrated in an fMRI study (Chen et al., 2006).

Music is rhythmically organized and unfolds in a predictable rhythmical structure enabling the listener to from expectations when upcoming musical events will occur. Although we did not find specific motor activation, it is conceivable that the short-term musical rhythm-focused training, that subjects had received prior to the MEG measurement, has established an internal forward model linking a piano tone with a specific motor movement. This internal forward model might have supported and improved predictions about the timing of upcoming tones. Expectation violations were than presumably processed in the parietal lobule where timing and the motor system are closely linked.

The dual pathway model of auditory processing has also been suggested by Hickok and Poeppel as an underlying mechanism for speech processing (2007). According to that model IPL, as part of the dorsal stream, is involved in sensorimotor mapping of sound to motor and articulatory networks. The ventral stream, on the other hand, is responsible for mapping sound to meaning. The dual stream model for language processing comprises thereby speech perception and production connecting the motor-speech-articulation systems in the parietal lobe with lexical-semantic representations (Hickok and Poeppel, 2007; Zaehle et al., 2008).

The beamformer analysis revealed that IFC activation was similar in the left and right hemispheres. This finding is consistent with our previous ERP analysis of the data. In contrast to our melody study, where we found a stronger involvement of the right hemisphere after a pitch deviation, the results of the rhythm study suggest that both hemispheres were involved when processing a rhythmic deviation. Previous studies showed that musical rhythm is processed bilaterally (Limb et al., 2006; Levitin, 2009; Lappe et al., 2011), or even left lateralized in professional musicians (Vuust et al., 2005).

In addition, the beamformer analysis revealed activation in the right insula, an area that is associated with auditory temporal processing (Bamiou et al., 2003). The right insula was shown to be involved in the detection of a temporal mismatch when subjects were asked to detect auditory-visual stimuli. The task activated the right insula, prefrontal cortex regions and also the IPL (Bushara et al., 2001). Although this is a cross-modal effect, there is evidence also from other studies that the insula plays a role in processing of temporal sequences (Griffiths et al., 1997).

## Conclusion

To investigate the neural generators of musical deviance detection for rhythmic and melodic material we compared beamformer localization of musical MMN data from two previous ERP studies, in which the musical MMN was measured after short-term piano training. We analyzed only post-training data, since in the pre-training data the MMN were not robust enough to conduct a beamformer analysis.

The training material of one study focused more strongly on the melodic harmonic progression whereas in the other study, training material focused on rhythmic progression. Accordingly, in the MEG measurements, subjects of the melody study were tested on a melody violation whereas subjects of the rhythm study were tested on a rhythmic violation.

In the melody study, the beamforming analysis revealed significant right hemispheric neural activation in the STG, IFC, and the SFG and OFG within a time window of 100–200 ms after the occurrence of a deviant tone. IFC and SFG activation was also observed in the left hemisphere. In contrast, in the rhythm study, the beamformer analysis revealed neural activation bilaterally within the vicinity of posterior auditory cortices and in the IPL, in an area that has previously been associated with temporal processing. The results are in line with studies investigating neural sources involved in harmonic or rhythmic deviance detection in musical material (Maess et al., 2001; Tillmann et al., 2003; Vuust et al., 2005). We conclude that different cortical networks are activated in the analysis of the temporal and the melodic content of musical material.

### Conflict of interest statement

The authors declare that the research was conducted in the absence of any commercial or financial relationships that could be construed as a potential conflict of interest.
